# Supplementary material for: Relationship between vitreous interleukin-6 levels and vitreous particles findings on widefield optical coherence tomography in posterior uveitis
Source: PLoS One. 2024 Jan 17;19(1):e0297201. doi: 10.1371/journal.pone.0297201 (PMC10793905; doi:10.1371/journal.pone.0297201)
Supplement: Attachment — Submitted filename: Rev_Relationship between vitreous interleukin-6 levels and vitreous particles findings on.docx [file pone.0297201.s002.docx]

3.11.2023

In this manuscript, the authors described “**Relationship between vitreous interleukin-6 levels and vitreous particles findings on widefield optical coherence tomography in posterior uveitis**”. The study will be very useful for the literature. The report is an interesting study, but it needed some suggestions for publication.

**Here are the concerns for the authors;**

**Generally;**

1. Keywords should be ordered alphabetical.
2. Grammar should be checked.
3. Abstract structure should be the same as described in the journal.
4. The inclusion and exclusion criteria determined for the study should be clearly stated in the materials and methods.
5. The findings given in the table should not be repeated in the text in the results section.
6. The units of evaluated parameters should be given clearly. For example, pg/mL for IL-6 value.
7. The % of demographic characteristics should also be given in Table 1.
8. The spelling features of Tables 1 and 2 are not the same.
9. All *p* values should be written in italic.
10. The resolution of the figures is low and should be corrected.
11. Citations should be corrected according to the journal instructions.
12. The lot number of kits should be stated.
13. The brand and model numbers of the devices used in the study should be stated.
14. What does the word “patiene” in Table 1 mean?
15. Significance levels of *p* values should be stated below the tables.
