# Supplementary material for: Relationship between vitreous interleukin-6 levels and vitreous particles findings on widefield optical coherence tomography in posterior uveitis
Source: PLoS One. 2024 Jan 17;19(1):e0297201. doi: 10.1371/journal.pone.0297201 (PMC10793905; doi:10.1371/journal.pone.0297201)
Supplement: Attachment — Submitted filename: renamed_e64f2.docx [file pone.0297201.s004.docx]

**#Reviewer1**

1. Line 204, line 353 and Fig 5. CD3 was examined in line 356 and Fig 5, whereas CD9 was examined in line 204. CD3 may be correct.

2. Line 344. In Table 4, there were no cases with ARN, thus “ARN acute retinal necrosis” should be erased.

**Response**

Thank you very much for your detailed corrections and advice. The above has been corrected.
